# Analytical validation of a circulating tumor DNA assay using PhasED-Seq technology for detecting residual disease in B-cell malignancies

**DOI:** 10.18632/oncotarget.28719

**Published:** 2025-05-09

**Authors:** Nina Klimova, Sandra Close, David M. Kurtz, Richard D. Hockett, Laura Hyland

**Affiliations:** ^1^Foresight Diagnostics, Boulder, CO 80301, USA

**Keywords:** MRD, ctDNA, PhasED-Seq, CLARITY, residual disease

## Abstract

Background: Circulating tumor DNA (ctDNA) can be used as a tool to detect minimal residual disease (MRD) which can provide important prognostic information in diffuse large B-cell lymphomas (DLBCL). Here, we present an ultra-sensitive MRD assay reliant on Phased Variant Enrichment and Detection Sequencing (PhasED-Seq), which leverages phased variants to detect ctDNA.

Methods: Blank plasma samples were used to assess assay specificity and a limiting dilution series with a DLBCL clinical-contrived sample was performed to assess assay sensitivity and precision. DLBCL plasma patient samples with MRD comparator assay results were tested with PhasED-Seq technology to assess assay accuracy.

Results: The assay’s false positive rate was 0.24% and the background error rate was 1.95E-08. The limit of detection at 95% detection rate with 120 ng of input DNA was 0.7 parts in 1,000,000 and precision was >96%. Positive percent agreement for the MRD assay was 90.62% (95% CI 74.98%, 98.02%) and negative percent agreement was 77.78% (95% CI 52.73, 93.59) using a single nucleotide variant-based method as reference.

Conclusions: The PhasED-Seq-based MRD assay has strong analytical and clinical performance in B-cell malignancies. Improved ctDNA detection methods such as this may improve patient outcomes through detection of residual disease or early relapse.

## INTRODUCTION

Circulating tumor DNA (ctDNA), tumor-derived DNA present in the bloodstream, is a non-invasive biomarker that can be used as a tool to detect minimal residual disease (MRD). Detection of cancer-specific somatic mutations from ctDNA can provide clinically relevant information to predict therapeutic response, disease recurrence, and survival, and thus guide intervention decisions [1–3]. As the utility of ctDNA detection has become appreciated, multiple investigational and commercially-available methods of detection have been developed [4]. However, the sensitivity of first-generation approaches is limited and improved methods are needed to detect residual ctDNA when tumor burden and ctDNA levels are low.

Diffuse large B-cell lymphoma (DLBCL) is the most common type of non-Hodgkin lymphoma (NHL) in the United States [5]. Despite attempts to increase the efficacy of conventional first-line immunochemotherapy over the past two decades, approximately 40% of DLBCL patients will have disease refractory to, or relapsing after, initial treatment [6]. Current DLBCL response criteria rely on functional radiographic indices such as positron emission tomography/computed tomography (PET/CT) scans, which have limited sensitivity and specificity [7]. There is a clear need to develop precision tools capable of rapid and accurate identification of patients harboring residual cancer burden who may be at high risk of relapse, such as the detection of residual tumor in the blood (i.e., ctDNA-MRD). A ctDNA-MRD platform has been developed based on Phased Variant Enrichment and Detection Sequencing (PhasED-Seq) to leverage phased variants (PVs) to improve the sensitivity of ctDNA detection compared to current approaches [8]. PVs are multiple somatic mutations in close proximity that can be concurrently observed on individual DNA molecules. PVs occur in most cancer types but are prevalent in stereotyped regions in B-cell malignancies [8], and are an attractive target to improve molecular detection techniques given their intrinsically low error profile [9]. This article describes the analytical validation of a sensitive PhasED-Seq-based ctDNA-MRD assay.

## RESULTS

### Quality control (QC) pass rate

The assay QC pass rate across all pre-analytic, analytic, and post-analytic metrics during the conduct of this analytical validation was 99.0%.

### Analytical specificity

The assay specificity was assessed using cell-free DNA (cfDNA) from 60 cancer-free donors (blank samples). All sample replicates passed QC metrics for 120 libraries for evaluation. Samples were sequenced to an average median depth of 21,321× and on-target coverage was greater than 91%. As blank samples do not have tumor-derived PVs, PV lists from 35 DLBCL patients were used to measure the false positive rate (FPR) and background error rate. The 35 PV lists covered 65.5% of the genomic regions targeted by the hybrid capture panel spanning multiple chromosomes. Each blank sample replicate was interrogated by 35 patient PV lists resulting in 4,200 possible tumor detection calls to evaluate the assay FPR (Supplementary Table 1). The overall assay FPR was 0.24%. The background error rate of the PhasED-Seq-based MRD assay was 1.95E-08, or 1.95 mutant molecules in 100 million informative molecules.

### Analytical sensitivity

**Figure 1:**
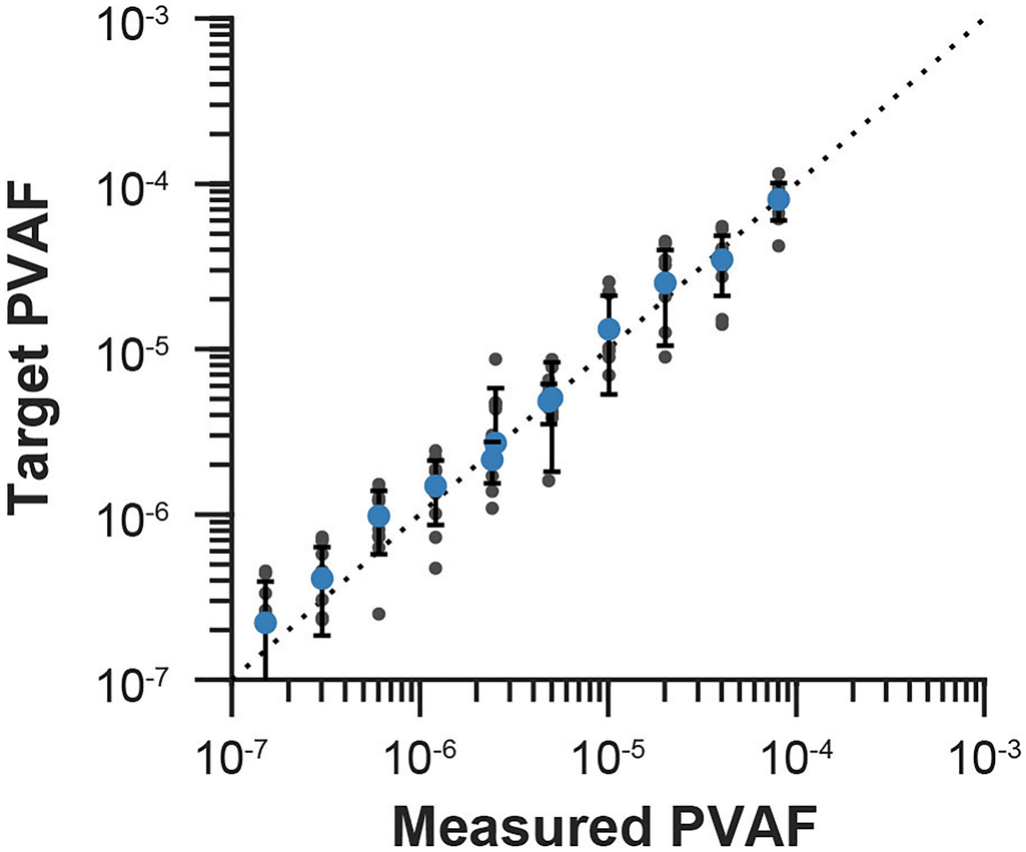
Limiting dilution series linearity. The measured PVAF for each replicate at each target PVAF dilution level created from the LoD clinical-contrived sample are plotted (black filled circles). Blue filled circles are the averages of the replicates at each target PVAF. The targeted PVAFs were calculated by using the average of the measured PVAF replicates for the highest dilution level and then using the dilution factors performed in study execution. For example, if a 1:2 dilution was performed, the new target PVAF is expected to be half of the previous dilution’s target PVAF. The dotted line represents a slope of 1 (i.e., x = y) and is shown for visualization. Figure shows test performance is as expected, i.e. when a 1:2 dilution is performed during study execution the measured PVAF also reflects this dilution.

DLBCL clinical-contrived sample replicates were prepared at 6 targeted phased variant allele fraction (PVAF) levels. All replicates passed QC metrics. Replicates were sequenced to an average median depth of 19,455×. The PV list generated for the clinical-contrived sample had a total of 9,043 PVs. In a dilution series ranging from 7.27E-04 to 1.51E-07, PVAF was linear with the dilution of mutant molecules (Figure 1). Detection rates at each PVAF level are presented in Table 1. Based on probit modeling the mutant molecules and PVAF corresponding to a detection rate of 95%, the limit of detection (LoD) of the MRD assay, is 3.11 mutant molecules and 6.61E-07 (Supplementary Figure 1), or 0.7 parts in 1,000,000.

**Table 1:** Analytical sensitivity

	Corrected PVAF level	*n*	*N*	Detection rate (%)
	4.83E-06	10	10	100
	2.42E-06	10	10	100
	1.21E-06	10	10	100
	6.04E-07	9	10	90
	3.02E-07	5	10	50
	1.51E-07	2	10	20
	0	0	4	0
**LoD95 PVAF - Probit**	6.61E-07			

Abbreviations: PVAF: phased variant allele fraction; *N*: total replicates at level; *n*: MRD positive results. Multiple lots were used to evaluate the detection rates at each target PVAF level.

### Reproducibility and repeatability

Table 2 shows the assay precision using clinical-contrived samples, covering 5 ng and 120 ng DNA input mass, and the low to high analytical measurement range. All sample replicates across operators, reagent lots, and timepoints (*N* = 104) passed QC metrics. Replicates were sequenced to an average median depth of 21,965×. Assay repeatability and reproducibility was greater than 96% (Table 2).

**Table 2:** DLBCL MRD assay precision results

	Input mass (ng)	Numerator (from APA equation)	Denominator (from APA equation)	APA (%)	95% Lower and Upper CI
Reproducibility	5	419	432.5	96.88	94.77, 98.3
Repeatability		60	62	96.77	79.19, 99.23
Reproducibility	120	149	149	100	97.55, 100
Repeatability		60	62	96.77	79.19, 99.23

Abbreviations: ng: nanograms; CI: confidence interval; APA: average positive agreement. Analyses were performed utilizing different lots, operators, and timepoints to generate maximum variability.

### Accuracy

Fifty samples from 19 individuals with DLBCL were evaluated for concordance between the PhasED-Seq-based MRD assay and a previously established single-nucleotide variant (SNV)-based method for MRD detection [10]. Clinical characteristics for all individuals are summarized in Supplementary Table 2. All samples passed QC metrics. Library input mass for plasma cfDNA samples for MRD detection ranged from 21.3 to 80 ng, and for non-cancerous, normal samples 80 ng of DNA was used. Normal samples were sequenced to an average median depth of 4,012× and cfDNA samples 5,980×. The number of PVs for each sample and donor was calculated and ranged from 1 to 1,816 PVs.

According to the comparator SNV-based method, 18 samples were called MRD negative and 32 were called MRD positive (Table 3). MRD monitoring samples that were called MRD positive by the comparator assay had a range of tumor fractions from 0.000022 to 0.1697. Using the PhasED-Seq-based MRD assay, 17 samples were called MRD negative and 33 samples MRD positive. The MRD positive samples as determined by the PhasED-Seq-based MRD assay had a range of PVAFs from 0.0000088 to 0.2567.

**Table 3:** Accuracy between-test concordance

		Comparator MRD Result		
		Negative (*n*)	Positive (*n*)	Total (*N*)
**PhasED-Seq-based MRD result**	**Negative (*n*)**	14	3	17
	**Positive (*n*)**	4	29	33
	**Total (*N*)**	18	32	50
	**NPA**	77.78% (95% CI 52.73%, 93.59%)		
	**PPA**	90.62% (95% CI 74.98%, 98.02%)		
	**OPA**	86.00% (95% CI 73.26%, 94.18%)		

Abbreviations: MRD: minimal residual disease; NPA: negative percent agreement; OPA: overall percent agreement; PhasED-Seq: Phased Variant Enrichment and Detection Sequencing; PPA: positive percent agreement.

Positive percent agreement (PPA) for the MRD assay was 90.62% (95% CI 74.98%, 98.02%) and negative percent agreement (NPA) was 77.78% (95% CI 52.73%, 93.59%; Table 3) using the SNV-based method as reference. There were 7 discordant calls between the two methods. In all the cases where the two assays were discordant (Table 4) the results of the PhasED-Seq-based MRD assay agreed with the clinical outcomes at each MRD monitoring timepoint and at end of therapy (EOT; PPA 100%, NPA 100%). In the same cases, the SNV-based comparator assay had a lower concordance with clinical outcomes (PPA 0%, NPA 60%).

**Table 4:** Discordant calls

Donor	Monitoring timepoint	PhasED-Seq-based MRD result	Comparator MRD result	MRD call discordance explanation
P1001	Baseline	NEGATIVE	POSITIVE	Patient cured with >5 years of follow-up and PET/CT scan with a Deauville Score of 1. PhasED-Seq-based MRD assay result matches clinical outcome. PhasED-Seq-based MRD assay result at EOT was MRD NEGATIVE.
P1001	Cycle2Day1	NEGATIVE	POSITIVE	Patient cured with >5 years of follow-up and PET/CT scan with a Deauville Score of 1. PhasED-Seq-based MRD assay result matches clinical outcome. PhasED-Seq-based MRD assay result at EOT was MRD NEGATIVE.
P1002	Cycle2Day1	POSITIVE	NEGATIVE	Blood collection was during treatment. Disease was still detected at Cycle2Day1, based on PhasED-Seq-based MRD assay result. Patient had additional cycles of therapy that likely cleared their disease. PhasED-Seq-based MRD assay result at Cycle3Day1 was MRD NEGATIVE which matches clinical outcome.
P1003	Cycle2Day1	POSITIVE	NEGATIVE	Blood collection was during treatment. Disease was still detected at Cycle2Day1, based on PhasED-Seq-based MRD assay result. Patient had additional cycles of therapy that likely cleared their disease. MRD assay result at EOT was PhasED-Seq-based MRD NEGATIVE which matches clinical outcome.
P1004	End of Therapy	NEGATIVE	POSITIVE	Patient cured with >5 years of follow-up and PET/CT scan with a Deauville Score of 1. PhasED-Seq-based MRD assay result at EOT matches clinical outcome.
P1005	Cycle3Day1	POSITIVE	NEGATIVE	Blood collection was during treatment. Disease was still detected at Cycle3Day1, based on MRD assay result. Patient had additional cycles of therapy that likely cleared their disease. MRD assay result at EOT was MRD NEGATIVE which matches clinical outcome.
P1006	Cycle3Day1	POSITIVE	NEGATIVE	Patient still has disease based on MRD assay result. MRD assay result at EOT was MRD POSITIVE which matches clinical outcome.

## DISCUSSION

The above describes the analytical validation of an MRD assay that utilizes PhasED-Seq technology to identify tumor-specific PVs to detect ctDNA in patients with B-cell lymphomas. The main benefit of using PVs for MRD detection is improving the signal-to-noise ratio in sequencing data by requiring the concordant detection of at least 2 separate non-reference events in an individual DNA molecule. Leveraging multiple somatic mutations within individual cfDNA fragments to detect ctDNA reduces the background error rate, as previously described [9]. The use of multiple variants on the same DNA strand (i.e., PVs) provides an advantage at low ctDNA levels in which the specificity of SNV-based technologies is reduced due to the inherent background error rate of SNVs.

The analyses presented here demonstrate the analytical performance of this PhasED-Seq-based MRD assay. The high specificity of the MRD assay was demonstrated through analysis of samples from individuals without cancer (*N* = 60), with a FPR of 0.24% and a background error rate of 1.95E-08, which is ~1,000-fold lower than reported for SNV-based technologies, even when utilizing unique molecular identifiers [9]. With this low background error rate, the analytical sensitivity of the PhasED-Seq-based MRD assay was determined to be 0.7 part per 1 million (6.61E-07 PVAF). This analytical sensitivity was determined using 120 ng of DNA and resulted in approximately 4 million informative molecules. The DNA input can be increased which will further increase the number of informative molecules and improve the analytical LoD due to the low background error rate.

Taken together, the assay’s high sensitivity and specificity suggests a reliable assay to detect low PVAFs without the accumulation of false-positive signal. At the increased sensitivity level, the assay’s reproducibility and repeatability rate was greater than 96% and proved to be robust to operator, reagent lot, and timepoint variability. Comparison of this PhasED-Seq-based MRD assay against an orthogonal SNV-based approach for detecting ctDNA-MRD demonstrated a high overall concordance. The discordant calls were adjudicated against patient clinical outcome data and for all discordant cases, the PhasED-Seq-based MRD assay correlated with clinical outcome.

Collectively, the data presented here suggest that the PhasED-Seq-based MRD assay is accurate and reproducible, making it appropriate for use in the clinical setting for individuals with B-cell malignancies. This is aligned with recent changes to the National Comprehensive Cancer Network guidelines to include ctDNA-MRD adjudication of positive PET results at the end of first line therapy [11]. Given the poor specificity of PET scans, the guidelines recommend additional testing to confirm residual lymphoma. Consistent with this, patients with detectable ctDNA-MRD are recommended to receive further treatment, while those without detectable ctDNA may follow the PET-negative pathway. Through the development of improved ctDNA detection methods such as that presented here, patient outcomes may be improved through the detection of residual disease or early relapse which may be used to guide treatment decisions.

## MATERIALS AND METHODS

### MRD assay overview

**Figure 2:**
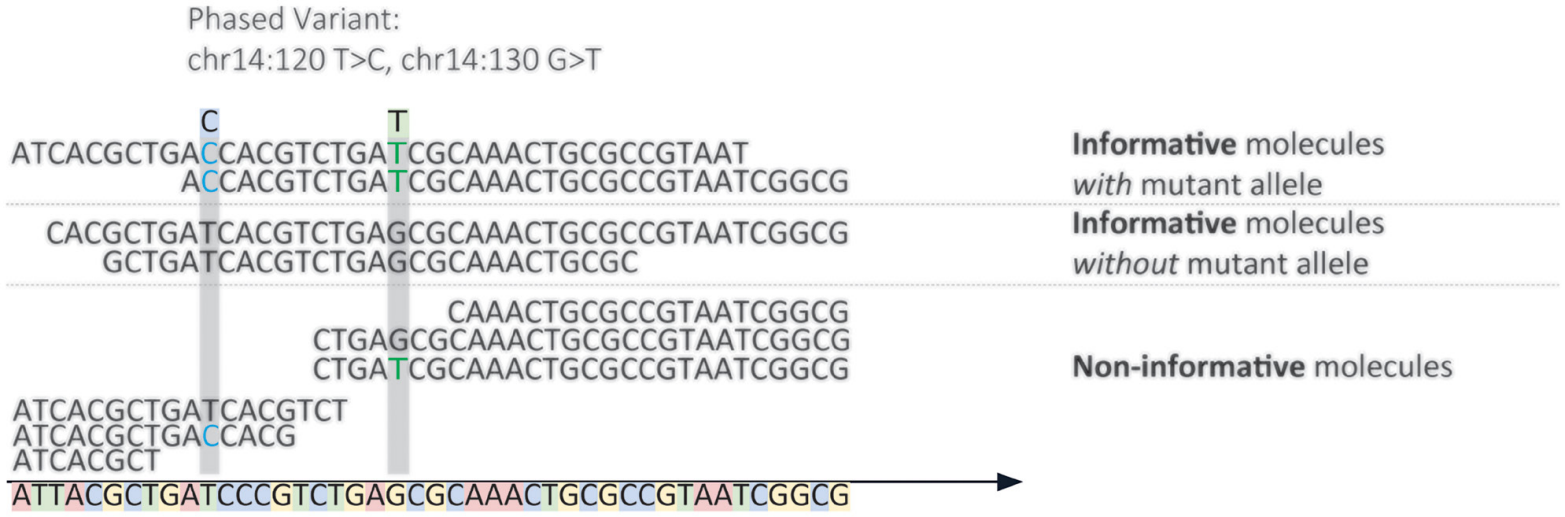
Depiction of informative molecules. ‘Informative Molecules’ are cfDNA molecules spanning the location of a tumor-specific PV. Any cfDNA molecules that could harbor a PV from the patient’s PV list are considered informative molecules. ‘Mutant molecules’ are informative molecules which harbor the tumor-specific mutant allele of one or more PVs in the patient’s PV list. In this example the PV chr14:120T>C, chr14:130G>T spans 4 informative molecules and 2 mutant molecules.

The Foresight CLARITY™ MRD assay (Foresight Diagnostics, Inc.; Supplementary Figure 2) was assessed. This assay utilizes a baseline DNA sample (pre-treatment plasma or tumor tissue), a non-cancerous or normal DNA sample (e.g., peripheral blood mononuclear cell (PMBC) germline DNA (gDNA)), and an MRD monitoring sample (e.g., plasma). Extracted DNA from all samples is sequenced using a fixed hybrid capture panel (~150 kb) that enriches for genomic regions in areas that recurrently harbor PVs in B-cell lymphomas. Following sequencing, PVs are identified in the tumor and non-cancerous DNA samples to generate a tumor-specific somatic PV list. Tumor-specific PVs are defined as those that are present in the tumor DNA sample and absent in the normal DNA sample. This tumor-specific PV list is then used to evaluate MRD monitoring sample(s) for the presence or absence of ctDNA using informative molecules. Informative molecules are cfDNA molecules spanning the location of a tumor-specific PV. Any cfDNA molecules that could harbor a PV from the patient’s PV list are considered informative molecules. Mutant molecules are informative molecules which harbor the tumor-specific mutant allele of one or more PVs in the patient’s PV List (Figure 2). MRD is defined as the presence of tumor-specific PVs (mutant molecules), meeting a threshold based on the likelihood of a non-somatic mutation overlapping with the tumor-specific PV list.

### Sample preparation

The analytical performance of the PhasED-Seq-based MRD assay was assessed in the Clinical Laboratory Improvement Amendments (CLIA)-registered laboratory at Foresight Diagnostics, Inc., following standard operating procedures. Samples included healthy donor samples (self-reported cancer-free at time of collection; *N* = 169; representative of the DLBCL patient population), clinical DLBCL samples (*N* = 76), and clinical-contrived DLBCL samples (*N* = 2). Clinical DLBCL samples were samples obtained from individuals with an active diagnosis of DLBCL. Clinical-contrived DLBCL samples were prepared by combining extracted cfDNA from clinical samples and healthy donor samples; multiple clinical DLBCL and healthy donor samples were pooled to make clinical-contrived samples. The clinical-contrived samples were then diluted to targeted PVAFs. PVAF is defined as the ratio of mutant molecules to informative molecules (e.g., the ratio of molecules containing a tumor-specific PV to total molecules spanning the positions of PVs). Both clinical and clinical-contrived samples were required to meet the following criteria for study inclusion: minimum input mass of 5 ng and ≥85% Phred quality score of 30 (Q30) from the Illumina sequencer. Sequencing metrics are reported within each study.

Both commercially procured (Discovery Life Sciences and BioIVT) and residual samples from academic research collaborations were utilized in the analytical validation studies. Samples from academic collaborations were collected with appropriate patient consent which allowed for research use of residual samples and institutional review board (IRB) oversight. Positive and negative controls were used along with each study sample batch. The positive control was a mix of lymphoma cell lines rich in PVs and the negative control consisted of libraries prepared with 50 μL nuclease-free water and carried through the entire workflow.

### DNA isolation

cfDNA was isolated from plasma using the QIAsymphony DSP Circulating DNA Kit (Qiagen, Hilden, Germany; Catalog Number: 937556) on the automated QIAsymphony system. Double-stranded (dsDNA) was quantified by fluorometry using a Qubit Fluorometer with the Qubit dsDNA High Sensitivity Assay Kit (Invitrogen, Waltham, MA, USA; Catalog Number: Q32854). gDNA was isolated from plasma-depleted whole blood (PDWB) or PBMCs using the commercial QIAsymphony DSP DNA Mini Kit (Qiagen, Hilden, Germany; Catalog Number: 937236) on the automated QIAsymphony system and sheared using sonication. DNA was quantified using the Qubit dsDNA Broad Range Assay Kit (Invitrogen, Waltham, MA, USA; Catalog Number: Q32853).

### Library preparation and next-generation sequencing

Library preparation, hybrid capture target enrichment, and sequencing by synthesis was performed according to Foresight Diagnostics, Inc., optimized workflows under standard operating procedures. Five to 120 ng of cfDNA or gDNA were used to construct sequencing libraries using KAPA HyperPrep Kits (Roche Sequencing Solutions, Indianapolis, IN) on manual and automated custom workflows. Library DNA was enriched using a custom B-cell lymphoma probe panel (Integrated DNA Technologies, Inc.), performed per the manufacturer’s instructions using both manual and automated custom workflows. Following enrichment, libraries were sequenced using sequencing by synthesis on the Illumina sequencing by synthesis technology (Illumina, San Diego, CA, USA).

### Analysis of sequencing data and MRD status determination

Sequence data were analyzed using in-house developed algorithms and pipelines. Briefly, raw sequencing data were demultiplexed to FASTQ files for each sample using BCL Convert software (Illumina, San Diego, CA; Versions 2.2.0 to 2.4.0). Low-quality sequencing reads and adapter read-through were removed using fastp (version 0.20.0). Sequencing reads were then aligned to the reference genome (GRCh37) using BWA-MEM aligner (version 2.2.1) to create one alignment file per sample, followed by proprietary methods to remove polymerase chain reaction (PCR) and optical duplicates. The resulting sequence alignment file was used for the analysis of PVs. MRD status was determined by the presence or absence of tumor-specific PVs, meeting a threshold based on the likelihood of a non-somatic mutation overlapping with the tumor-specific PV list.

### Analytical specificity

The assay specificity or limit of blank (LoB) was evaluated according to CLSI guidance EP17-A2 [12]. EP17-A2 defines the LoB as the highest value expected to be observed from a series of measurements on a sample that contains no analyte (blank samples). Whole blood from 60 cancer-free donors (blank samples) was collected in Streck cfDNA blood collection tubes (BCTs; Streck, Catalog Number: 230470), processed to plasma, and cfDNA. DNA input mass into library preparation was 120 ng, corresponding to the upper limit for DNA input for the assay. Two library replicates were prepared from each donor and a total of 120 libraries were generated for sequencing. Libraries were interrogated by DLBCL tumor-specific PV lists resulting in a MRD positive or negative call. The FPR and background error rate for the assay was calculated per donor and overall.

### Analytical sensitivity

To determine the LoD of the MRD assay (95% detection rate per CLSI EP17-A2), a limited dilution series of a DLBCL clinical-contrived sample was prepared at 6 targeted PVAF levels (7.00E-06, 3.50E-06, 1.75E-06, 8.75E-07, 4.38E-07, and 2.19E-07). Clinical-contrived sample replicates were created by combining cfDNA from 4 DLBCL patient samples and diluting the mixture into background cfDNA from healthy donor plasma. Ten replicates were tested across 2 reagent lots.

Per CLSI EP17-A2, probit modeling was used to evaluate the LoD [12]. Corrected targeted PVAF levels, based on the observed PVAFs, were used for detection rate and probit models. The detection rate for each level was calculated by adding the number of MRD positive calls and dividing the number by the total number of replicates tested. The probit model was used to compute the number of mutant molecules and PVAF corresponding to a detection rate of 95% for the sample. Probit model fit was acceptable by evaluating with a statistical goodness of fit test.

### Reproducibility and repeatability

Assay precision was evaluated with a clinical-contrived MRD positive sample that was prepared across different targeted PVAF levels. At 120 ng input mass, targeted PVAF levels were 7.00E-06, 3.50E-06, and 1.75E-06 and at 5 ng input mass targeted PVAF levels were 1.00E-4, 4.00E-5, and 2.00E-5. Average positive agreement (APA) was used to calculate the assay’s repeatability and reproducibility as described in Yu et al, 2016 [13]. Sample replicates were prepared across 2 operators, 2 reagent lots, and 3 timepoints.

### Accuracy

The accuracy of the PhasED-Seq-based MRD assay was determined by comparing the results to a previously established SNV-based orthogonal method for detection of ctDNA using samples from individuals with DLBCL [10]. Samples from a total of 19 individuals with DLBCL were utilized, including 19 pre-treatment plasma samples, 19 normal samples, 31 plasma samples from timepoints of interest during treatment (Cycle 2 Day 1 or Cycle 3 Day 1) or at EOT. MRD testing with the PhasED-Seq-based MRD assay was performed blinded to clinical outcomes. Results from the SNV-based MRD assay and the PhasED-Seq-based MRD assay were compared. Discordant results between the two assays were adjudicated by comparison with clinical outcomes.

## SUPPLEMENTARY MATERIALS



## Abbreviations

APA: average percent agreement
BCTs: blood collection tubes
cfDNA: cell-free DNA
ctDNA: circulating tumor DNA
DLBCL: diffuse large B-cell lymphoma
EOT: end of therapy
FPR: false-positive rate
gDNA: germline DNA
LoB: limit of blank
LoD: limit of detection
MRD: minimal residual disease
NHL: non-Hodgkin lymphoma
NPA: negative percent agreement
PBMCs: peripheral blood mononuclear cells
PCR: polymerase chain reaction
PDWB: plasma-depleted whole blood
PET/CT: positron emission tomography/computed tomography
PhasED-Seq: Phased Variant Enrichment and Detection Sequencing
PPA: percent positive agreement
PV: phased variant
PVAF: phased variant allele fraction
QC: quality control
SNV: single nucleotide variant
