# Supplementary material for: Analytical validation of a circulating tumor DNA assay using PhasED-Seq technology for detecting residual disease in B-cell malignancies
Source: Oncotarget. 2025 May 9;16:329–36. doi: 10.18632/oncotarget.28719 (PMC12068320; doi:10.18632/oncotarget.28719)
Supplement: Supplementary file 1 [file oncotarget-16-28719-s001.pdf]

Analytical validation of a circulating tumor DNA assay using PhasED-Seq technology for detecting residual disease in B-cell malignancies

SUPPLEMENTARY MATERIALS

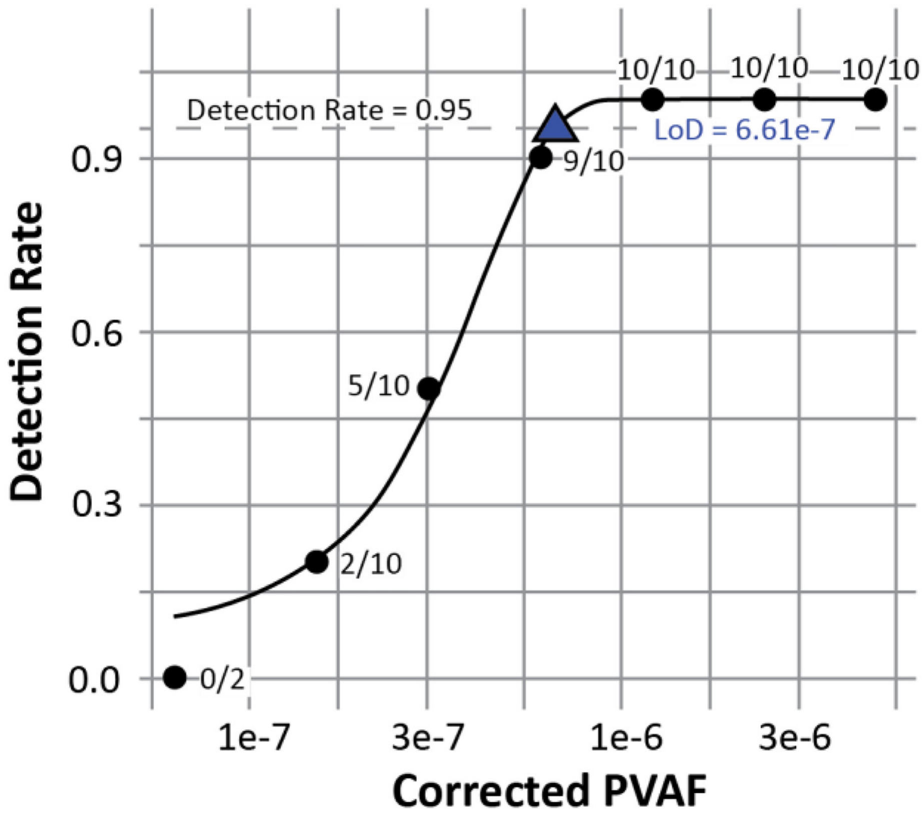

Supplementary Figure 1: PROBIT Graph. The relationship between detection rate and PVAF is modeled by probit model regression. The black dots represent the observed data points while the solid black line represents probit model's predict probabilities. The detection rates at the specific PVAF levels are presented as the number of MRD positive calls/total number of replicates. The dotted line represents the detection rate at 0.95 (95%). Blue text is the PVAF at 95% detection rate for the sample or the limit of detection (LoD).

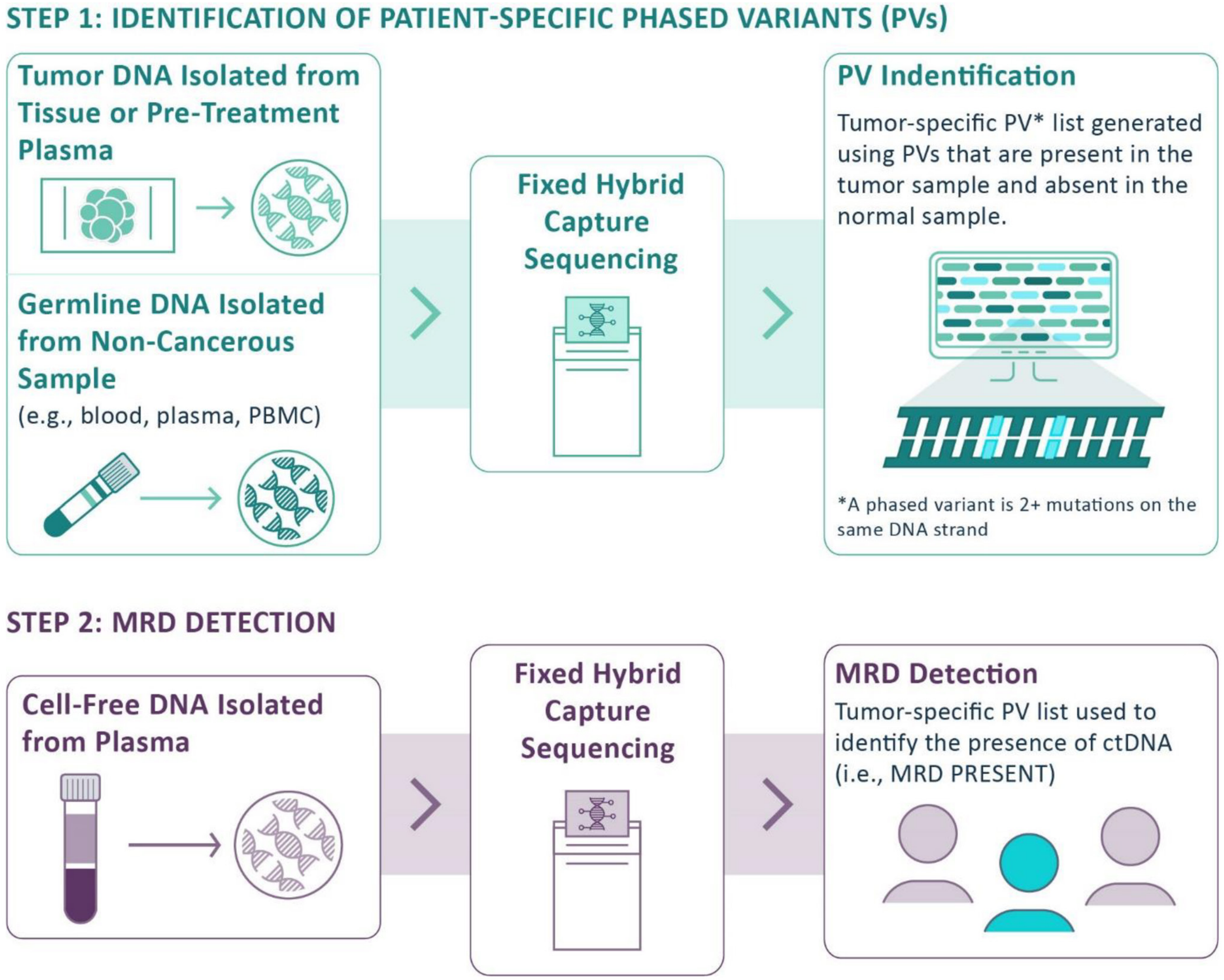

Supplementary Figure 2: Overview of foresight CLARITY MRD assay process. The PhasED-Seq-based MRD assay involves two steps: identification of tumor-specific phased variants followed by MRD detection. Abbreviations: ctDNA: circulating tumor DNA; PBMC: peripheral blood mononuclear cells; PVs, phased variants.

Supplementary Table 1: Overall background error rate

Donor	# replicates	# PV lists interrogated	Mutant molecules	Informative molecules	Background error rate (fraction)	Total MRD positive calls	Total expected MRD negative calls	False positive rate (%)
1	2	35	5	98,793,024	5.06E-08	0	70	0
2	2	35	1	77,767,440	1.29E-08	1	70	1.43
3	2	35	1	98,627,955	1.01E-08	0	70	0
4	2	35	1	86,919,492	1.15E-08	0	70	0
5	2	35	1	107,266,708	9.32E-09	0	70	0
6	2	35	2	100,645,424	1.99E-08	0	70	0
7	2	35	1	96,817,244	1.03E-08	0	70	0
8	2	35	3	78,652,048	3.81E-08	0	70	0
9	2	35	0	106,243,184	0	0	70	0
10	2	35	2	117,732,313	1.70E-08	0	70	0
11	2	35	0	118,474,936	0	0	70	0
12	2	35	1	103,088,419	9.70E-09	0	70	0
13	2	35	1	118,450,145	8.44E-09	0	70	0
14	2	35	1	95,051,106	1.05E-08	0	70	0
15	2	35	5	114,203,030	4.38E-08	0	70	0
16	2	35	1	108,918,282	9.18E-09	0	70	0
17	2	35	2	92,932,858	2.15E-08	0	70	0
18	2	35	2	101,447,740	1.97E-08	0	70	0
19	2	35	4	113,345,578	3.53E-08	0	70	0
20	2	35	1	100,449,094	9.96E-09	0	70	0
21	2	35	0	106,473,194	0	0	70	0
22	2	35	1	111,129,428	9.00E-09	0	70	0
23	2	35	6	94,954,187	6.32E-08	1	70	1.43
24	2	35	0	114,461,552	0	0	70	0
25	2	35	4	95,107,444	4.21E-08	0	70	0
26	2	35	3	106,489,001	2.82E-08	0	70	0
27	2	35	0	92,476,518	0	0	70	0
28	2	35	0	100,074,791	0	0	70	0
29	2	35	0	101,027,578	0	0	70	0
30	2	35	1	97,201,850	1.03E-08	0	70	0
31	2	35	2	90,765,906	2.20E-08	0	70	0
32	2	35	2	100,148,187	2.00E-08	1	70	1.43
33	2	35	4	96,188,440	4.16E-08	0	70	0
34	2	35	0	97,427,939	0	0	70	0

35	2	35	6	103,362,518	5.80E-08	1	70	1.43
36	2	35	3	85,663,958	3.50E-08	0	70	0
37	2	35	2	80,566,388	2.48E-08	1	70	1.43
38	2	35	2	105,450,456	1.90E-08	0	70	0
39	2	35	1	91,265,495	1.10E-08	0	70	0
40	2	35	4	82,989,868	4.82E-08	0	70	0
41	2	35	2	76,568,244	2.61E-08	0	70	0
42	2	35	1	103,160,223	9.69E-09	0	70	0
43	2	35	0	108,668,963	0	0	70	0
44	2	35	2	90,103,243	2.22E-08	0	70	0
45	2	35	3	95,641,814	3.14E-08	0	70	0
46	2	35	2	101,762,522	1.97E-08	1	70	1.43
47	2	35	4	115,434,670	3.47E-08	0	70	0
48	2	35	3	101,580,713	2.95E-08	0	70	0
49	2	35	2	93,769,944	2.13E-08	0	70	0
50	2	35	2	112,613,443	1.78E-08	1	70	1.43
51	2	35	0	86,935,981	0	0	70	0
52	2	35	4	102,154,285	3.92E-08	0	70	0
53	2	35	1	63,890,656	1.57E-08	0	70	0
54	2	35	2	76,800,239	2.60E-08	1	70	1.43
55	2	35	2	78,998,788	2.53E-08	1	70	1.43
56	2	35	2	83,356,343	2.40E-08	0	70	0
57	2	35	3	91,487,653	3.28E-08	0	70	0
58	2	35	0	90,276,663	0	0	70	0
59	2	35	2	111,065,706	1.80E-08	0	70	0
60	2	35	2	121,232,606	1.65E-08	1	70	1.43
**Overall**	**120**	**35**	**115**	5,894,553,417	**1.95E-08**	**10**	**4200**	**0.24**

Supplementary Table 2: Clinical characteristics for samples used in analytical accuracy

**Age at diagnosis (Median, Range)**		**55 (21–80)**
**Gender**	Female	6/19 (32%)
	Male	13/19 (68%)
**Stage**	1	1/19 (5.3%)
	2	3/19 (16%)
	3	3/19 (16%)
	4	12/19 (63%)
**IPI**	0	2/19 (11%)
	1	6/19 (32%)
	2	4/19 (21%)
	3	4/19 (21%)
	4	2/19 (11%)
	5	1/19 (5.3%)
**Cell of origin**	GCB	11/19 (58%)
	unknown	8/19 (42%)
**Treatment**	CODOX-M	1/19 (5.3%)
	EPOCH-R	11/19 (58%)
	R-CHOP	7/19 (37%)
**Best response (PET/CT)**	CR	15/19 (79%)
	PD	1/19 (5.3%)
	PR	2/19 (11%)
	SD	1/19 (5.3%)
**Progression event (Yes/No)**	No	12/19 (63%)
	Yes	7/19 (37%)
**PFS duration (months; median, range)**		46 (1-128)
**Deauville score**	1	6/19 (32%)
	2	1/19 (5.3%)
	3	6/19 (32%)
	4	3/19 (16%)
	5	1/19 (5.3%)
	N/A	2/19 (11%)

PFS duration indicates the time to event or the amount of follow-up, in the case of no progression event. EPOCH-R includes m-it da-EPOCH-R and DA-EPOCH-R. R-CHOP includes R-miniCHOP. Abbreviations: CR: complete response; GCB: germinal center B-cell-like; IPI: international prognostic score; PD: progressive disease; PFS: progression-free survival; PR: partial response; SD: stable disease.
